# Molecular characterization and antibiotic resistance patterns of avian fecal *Escherichia coli* from turkeys, geese, and ducks

**DOI:** 10.14202/vetworld.2018.859-867

**Published:** 2018-06-27

**Authors:** Nokukhanya Dube, Joshua Mbanga

**Affiliations:** Department of Applied Biology and Biochemistry, Faculty of Applied Sciences, National University of Science and Technology, Bulawayo, Zimbabwe

**Keywords:** antibiotic resistance, avian fecal *Escherichia coli*, poultry, virulence gene

## Abstract

**Background and Aim:**

Avian fecal *Escherichia coli* (AFEC) are considered to be the natural reservoir of pathogenic strains in extraintestinal infections as such characterization of AFEC gives insight into the spread of the potential pathogenic lineage. The aim of the study was to investigate the reservoirs of avian pathogenic *E. coli* (APEC) from fecal samples of healthy ducks, geese, and turkeys by determining the antibiotic resistance patterns of AFEC isolates from turkeys, geese and ducks and characterization of the isolates using virulence genes, plasmid profiles, and phylogenetic grouping.

**Materials and Methods:**

The disc diffusion method was used to determine antibiotic resistance of 100 AFEC isolates from turkeys (9), geese (29), and ducks (62) to 8 antibiotics. Molecular characterization of the isolates was done by multiplex polymerase chain reaction to investigate the presence of 12 virulence genes, plasmid profiling, and phylogenetic grouping based on the 16S rRNA sequences.

**Results:**

Antibiogram profiles indicated maximum resistance to cloxacillin (100%) and bacitracin (100%) for all AFEC isolates and high sensitivity to ciprofloxacin; however, all isolates exhibited multi-drug resistance. The AFEC isolates from turkeys (6) and geese (12) did not contain virulence genes. The *frz* (3.7%), *sitD* (29.6%), and *fimH* (92.5%) were detected in the duck isolates. None of the isolates had the *KpsM*, *iutA*, *vat*, *sitA*, *hlyF*, *pstB*, *ompT*, *uvrY*, and *sopB* genes. Plasmid profiling gave four plasmid profiles with the plasmids ranging from 1.5 to 55 kb. Phylogenetic analysis of 16S rRNA sequences revealed similarities between AFEC isolates from the different poultry species, as the isolates did not cluster according to avian species.

**Conclusion:**

AFEC isolates are potential reservoirs of APEC as they contain some of the virulence genes associated with APEC. Multidrug resistance is high in AFEC isolated from healthy birds. This is a public health concern.

## Introduction

*Escherichia coli* is a member of the *Enterobacteriaceae* and is a well-known ubiquitous member of the normal intestinal bacterial microflora in warm-blooded animals [1,2]. Most strains of *E. coli* are harmless and are considered as commensal *E. coli* [1]. Normally *E. coli* persists as a harmless commensal in the mucous layer of the cecum and colon and has adapted its metabolism very successfully to the nutritional ecological niche [1,3]. Commensal *E. coli* has been shown to be quite diverse and may contain different fitness traits which are considered reservoirs of virulence traits. In addition, the avian fecal *E. coli* (AFEC) have shown a nonrandom distribution of several plasmid replicon types [3]. Although most *E. coli* strains belong to the normal flora of the intestines and are nonpathogenic, there are some strains that are able to establish themselves outside of the intestines and cause diseases, these fall into the extraintestinal pathogenic *E. coli* (ExPEC) [3]. AFEC show great variability owing to a marked genome plasticity. An unambiguous distinction of extraintestinal pathogenic and AFEC is not easy as the strains with the ability to cause extraintestinal infections are facultative pathogens and belong to the normal flora of healthy birds. AFEC are considered to be the natural reservoir of pathogenic strains in extraintestinal infections as such characterization of AFEC gives insight into the spread of the potential pathogenic lineage [4]. Commensal *E. coli* possess traits that are largely similar in function to virulence traits. The presence of adhesins, capsular antigens, and iron acquisition system is positively correlated to the time of their persistence in the intestinal regions and enables AFEC to be capable of long-term intestinal colonization [2,5]. The presence of fitness traits, though key to commensality, promotes bacterial adhesion in niches which are extraintestinal [1]. ExPEC share virulence attributes which enable their extraintestinal lifestyle [6]. A variety of virulence factors have been investigated, these include adhesins, hemolysins, iron acquisition systems, antibactericidal factors, and toxins [7-9]. More commonly reported virulence factors associated with avian pathogenic *E. coli* (APEC) are increased serum survival, production of aerobactin, K1 capsule, presence of type 1 and P fimbriae and temperature sensitive hemaglutinin [10,11]. However, APEC strains are very diverse and their diversity is related to the diversity of their virulence factors and serotypes [12]. Although several virulence factors exist, the virulence factors have been shown to be rarely all present in the same isolate and that they can occur individually or polygenically [13].

Antibiotic resistance is a global problem affecting both human and veterinary medicine, and an increase in resistance against antibiotics renders control of infections difficult. Antibiotics have been used in the poultry industry for various uses such as treatment of infections and as growth promoters and the situation has been exacerbated by the emergence of multidrug-resistant strains. The overuse of antibiotics results in intestinal flora being consistently exposed to antibiotics and ultimately in the acquisition of antibiotic resistance mechanisms [14]. Limited studies have been carried out globally on the emergence of reservoir organisms that have acquired multidrug-resistant (MDR) as well as pathogenic traits. It has thus proved difficult to distinguish between AFEC and APEC.

Although several studies are showing extensive research on the APEC there remains a gray area on the reservoirs of APEC which needs to be clearly defined, and little work has been in done in Africa on the differentiation of AFEC and APEC using virulence genotyping. The study sought to investigate the reservoirs of APEC from fecal samples of healthy ducks, geese, and turkeys by determining the antibiotic resistance patterns of AFEC isolates from turkeys, geese, and ducks and characterization of the isolates using virulence genes, plasmid profiles, and phylogenetic grouping.

## Materials and Methods

### Ethical approval

No Ethical approval was required although all applicable international, national, and institutional guidelines for the care and use of animals were followed.

### Sample collection

A total of 100 fecal samples were collected from the cloaca of 100 healthy poultry using sterile swabs. The samples were collected from 9 turkeys, 29 geese, and 62 ducks from a poultry farm in Bulawayo, Zimbabwe. All samples were from adult birds except for 13 samples which were obtained from 10 ducklings and 3 poults. The poultry farm practices a “free range system” was the poultry are allowed to roam outdoors during the day and intermingle freely. They are, however, locked in cages overnight.

### Isolation and identification of *E. coli*

The swabs were dipped in sterile 0.8% saline solution before 100 µl of the sample being cultured on 5% sheep blood agar (New England Biolabs, South Africa) and incubated aerobically at 37°C for 24 h. Suspected *E. coli* colonies were then incubated on MacConkey agar (Sigma-Aldrich, United Kingdom) and Eosin-Methylene Blue agar (Oxoid, U.K) plates and incubated as above. The identification of *E. coli* was done according to methods described by Barrow and Feltham [15]. Biochemical tests included the Gram stain and the catalase, oxidase, indole, citrate, methyl red, and Voges–Proskauer tests.

### Antimicrobial susceptibility testing

The Kirby-Bauer disc diffusion assay was used to test the susceptibility of the AFEC isolates on Mueller-Hinton agar (Oxoid, Basingstoke, UK). Each isolate was tested for antibiotic susceptibility using a panel of the following antibiotics: ampicillin (25 µg), bacitracin (10 µg), ciprofloxacin (5 µg), chloramphenicol (30 µg), cloxacillin (5 µg), gentamicin (10 µg), nalidixic (30 µg), and neomycin (10 µg). All antibiotics were from Oxoid. The plates were incubated at 37°C for 24 h and the zones of inhibition measured. The results were interpreted according to Clinical Laboratory Standards Institute (2014) guidelines. *E. coli* reference strain ATCC 25922 was used as the reference strain in all tests.

### DNA extraction

Bacterial strains were subcultured at 37°C overnight in nutrient broth (Oxoid, Basingstoke and Hampshire, UK) and genomic DNA was extracted using a standard Phenol-Chloroform method by Sambrook and Russell [16]. The purity of DNA was checked by running a 1% ethidium bromide-stained agarose gel (Sigma-Aldrich, St. Louis, USA) with a 1 kb DNA ladder (Thermo Scientific, USA) in tris-borate-EDTA (TBE) buffer for 1 h at 100 V and then viewed using a Uvipro-Silver Gel Documentation System (Uvitec, UK). The concentration of DNA was determined using a Qubit 3.0 fluorometer (Thermo Scientific, USA).

### Virulence genotyping

The presence of genes encoding virulence factors was detected using multiplex polymerase chain reaction (PCR) amplification. Four multiplex PCR assays were used to detect 12 virulence genes (Table-S1). The multiplex design was according to that reported by van der Westhuizen and Bragg [17] with slight changes in the primer and final magnesium chloride concentrations. The effected changes were using primer concentrations of 0.5 µM for the *frz*, *sitD*, *fimH*, *OmpT*, *iutA*, *pstB*, and *SopB* genes and adjusting the final MgCl_2_ concentration to 3 mM for all multiplex reactions. The primers used in our study have been reported in previous studies [13,17]. All primers used were obtained from Inqaba Biotech, South Africa. Three microliters of each of the DNA samples were mixed with all necessary components for amplification in a 0.2 ml PCR tube (Perkin-Elmer, USA) in a 25 µl reaction. The reaction mixture included 2.5 µl of 10× PCR Dream Taq buffer (Thermo Scientific, USA), 2 µl of dNTPs, 10 mM; 0.25 µl of Dream *Taq* polymerase (Thermo Scientific, USA), 5 U/µl, and nuclease-free water to maintain a total volume of 25 µl. The appropriate primers ranging from 0.5 µM to 2 µM were added, and the MgCl_2_ concentration was adjusted to a final concentration of 3 mM. Negative controls comprised of water control. An Applied Biosystems GeneAmp^®^ PCR System 9700 was used for the PCR thermal cycling conditions with an initial denaturation step at 94°C for 5 min, 35 cycles (denaturation 94°C for 30 s, annealing at 63°C for 45 s, extension 72°C for 1 min and 45 s) and a final elongation step at 72°C for 10 min. The amplified products were then run on a 1% ethidium bromide-stained agarose gel with a 100 bp DNA ladder (Thermo Scientific, USA) in TBE buffer for 1 h at 100 V and then viewed using Uvipro-Silver Gel Documentation System (Uvitec, UK). The multiplex PCR was used to screen for 12 virulence genes. The presence or absence of virulence genes was used to calculate the prevalence of virulence genes and assign virulence gene profiles as shown in Table-1.

**Table-S1:** Final primer concentrations used in the different multiplex PCRs.

Multiplex	Primer set	Concentration (µM)	Primer set	Concentration (µM)	Primer set	Concentration (µM)	Additional MgCl_2_ (mM)	Final MgCl_2_ (mM)
1	*frz*	0.5	*sitD*	0.5	*fimH*	0.5	1	3
2	*sitA*	2.0	*kpsM*	1.0	*Vat*	0.5	1	3
3	*ompT*	0.5	*iutA*	0.5	*pstB*	0.5	1	3
4	*sopB*	0.5	*uvrY*	1.0	*hlyF*	0.5	1	3

PCR=Polymerase chain reaction

**Table-1:** Percentage frequency of virulence genes in AFEC from ducks.

Name of gene	Primer	Frequency
Frz operon	*Frz*	3.7%
*Sit*ABCD system	*SitA*	0%
*Sit*ABCD	*SitD*	29.6%
Type 1 fimbrial adhension	*FimH*	92.3%
Capsular protein transport of polysaccharides	*KpSM*	0%
Vacuolating autotransporter	*Vat*	0%
Episomal outer membrane protease	*OmpT*	0%
Aerobactin siderophore	*IutA*	0%
*Pst*SCAB system	*pSTB*	0%
Plasmid partitioning protein	*sopB*	0%
Putative avian hemolysin	*HlFy*	0%
APEC virulence regulator	*Uvry*	0%

AFEC=Avian fecal *Escherichia coli*

### Plasmid profiling

Plasmid DNA isolation was done using the Zyppy^™^ miniprep kit (Zymo Research, UK). The plasmid DNA together with DNA ladder was run on a 0.8% agarose gel stained with ethidium bromide. The bands were visualized using the Uvipro Silver Gel Documentation System (Uvitec, Cambridge, UK).

### Amplification of 16S ribosomal DNA (rDNA)

The 16S rDNA was amplified using PCR by making use of the primers 27F (5’-AGAGTTTGATCCTGGCTCAG-3’) and 1492R (5’-GGTTACCTTGTTACGACTT-3’). The 25 µl PCR reaction consisted of 5× One Taq standard PCR buffer, 10 mM dNTPs, 10 µM of each primer, One Taq DNA polymerase (Biolabs Scientific, USA) and DNA template. The PCR cycle started by an initial denaturation of 94°C for 30 s, 30 cycles of 94°C for 30 s, 60°C for 30 s, and 68°C for 1 min 30 s. Final extension was done at 68°C for 5 min and samples at 4°C.

### Sequencing of PCR products

A total of 33 PCR products AFEC ducks (19), AFEC geese (8), and AFEC Turkeys (6) were sent for sequencing. The number was determined by financial limitations. Sequencing of purified PCR products was performed at Inqaba Biotech, Pretoria, South Africa, using an automated ABI3500XL Genetic Analyser and BigDye terminator v3.1 cycle sequencing reactions (Applied Biosystems, Foster City, CA) according to the manufacturer’s instructions. DNA data (chromatographs and sequences) were sent back by email for analysis. The same primers used to carry out the amplification of the 16S rRNA gene in the laboratory were used in the sequencing reactions. The sequences were supplied in the form of ab1 files, and the sequence analysis was done using Basic Local Alignment Search Tool in the NCBI databases. Phylogenetic analysis was done using Molecular Evolutionary Genetics Analysis version 7 (MEGA7) [18].

## Results

From the 100 birds sampled in the study, a total of 54 *E. coli* isolates were obtained. The distribution of AFEC in the different types of birds was noted, and the prevalence values were calculated. In total, 35/62 (56%) AFEC were obtained from ducks, 12/29 (41%) from geese, and 7/9 (78%) from turkeys.

### Antibiotic resistance

**Figure-1:**
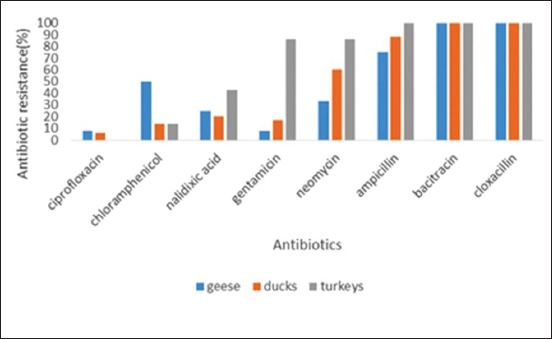
Antibiogram of avian fecal *Escherichia coli* from turkeys, geese, and ducks.

The 54 AFEC isolates were tested for their susceptibility to eight antimicrobial agents using the Kirby-Bauer disc diffusion assay. All isolates were resistant to bacitracin and cloxacillin. All isolates were highly susceptible to ciprofloxacin, turkey AFEC isolates showed 100% susceptibility, duck isolates, 88% and geese isolates, 84% (Figure-1).

The AFEC isolates exhibited multidrug resistance as they were resistant to two or more antibiotics.

The analysis of the antibiotic resistance patterns revealed 17 antimicrobial resistance patterns (Table-2).

**Table-2:** Antimicrobial resistance patterns for AFEC isolates.

Antimicrobials pattern	Number of resistant isolates			Pattern

	Ducks	Turkey	Geese	
Amp Gm Cx Ba	2	1		A
Amp Gm Cx Ne Ba	2	2		B
Amp Gm Cx Ne Ba C		1		C
Amp Cx Ne Ba Na	3	1		D
Amp Gm Cx Ne Ba Na		2		E
Cx Ba	3		2	F
Cx Ne Ba	1		1	G
Amp Cx Ba	6		4	H
Amp Cx Ba C	1		1	I
Gm Cx Ne Ba	1			J
Amp Cx Ne Ba	9		1	K
Amp Cx Ne Ba C	3		1	L
Amp Cip Cx Ne Ba	1			M
Amp Cip Cx Ba C	1			N
Amp Cx Ne Ba C Na	1			O
Amp Gm Cx Ba C Na	1			P
Cx Ne Ba C			1	Q

Amp=Ampicillin, Cip=Ciprofloxacin, Ne=Neomycin, Cx=Cloxacillin, Ba=Bacitracin, Na=Nalidixic acid, C=Chloramphenicol, Gm=Gentamicin, AFEC=Avian fecal *Escherechia coli*

### Virulence genotyping

In virulence genotyping, 45 isolates from turkeys (6), geese (12), and ducks (27) were used. After successful DNA isolation and quantification, the DNA of each of the 45 AFEC isolates was subjected to four different multiplex PCRs to investigate the presence of 12 virulence genes associated with APEC. Each multiplex reaction amplified three APEC-associated virulence gene regions. The isolates from turkeys and geese did not contain any virulence genes. Of the 12 genes investigated only three virulence genes were detected in the duck isolates, *frz* (3.7%), *sitD* (29.6%) and *fimH* (92.5%). Table-3 shows the results for the duck isolates only. The virulence genes detected in ducks AFEC isolates were for multiplex set A, which targeted the *frz*, *fimH*, and *sitD* genes only. Only one isolate had three genes, and of the 45 isolates examined for virulence genes, 44 isolates exhibited profile A (had 0-2 genes) and only 1 isolate exhibited profile B (3-5 genes).

**Table-3:** Virulence genes detected in AFEC from ducks.

Isolate	Multiplex A			Multiplex B			Multiplex C			Multiplex D			Total	Profile
			
	*Frz*	*sitD*	*fimH*	*SitA*	*KpSM*	*Vat*	*OmpT*	*IutA*	*PsTB*	*SopB*	*UvrY*	*Hlfy*		
4DA	-	-	+	-	-	-	-	-	-	-	-	-	1	A
7DA	-	-	+	-	-	-	-	-	-	-	-	-	1	A
12DA	-	-	+	-	-	-	-	-	-	-	-	-	1	A
15DA	-	-	+	-	-	-	-	-	-	-	-	-	1	A
23DA	-	+	+	-	-	-	-	-	-	-	-	-	2	A
23DY	-	+	+	-	-	-	-	-	-	-	-	-	2	A
26DA	-	-	+	-	-	-	-	-	-	-	-	-	1	A
27DY	-	-	+	-	-	-	-	-	-	-	-	-	1	A
31DY	-	-	+	-	-	-	-	-	-	-	-	-	1	A
32DA	+	+	+	-	-	-	-	-	-	-	-	-	3	B
33DA	-	-	+	-	-	-	-	-	-	-	-	-	1	A
34DA	-	+	+	-	-	-	-	-	-	-	-	-	2	A
35DA	-	+	+	-	-	-	-	-	-	-	-	-	2	A
37DA	-	-	+	-	-	-	-	-	-	-	-	-	1	A
39DA	-	-	+	-	-	-	-	-	-	-	-	-	1	A
46DA	-	-	+	-	-	-	-	-	-	-	-	-	1	A
48DA	-	-	+	-	-	-	-	-	-	-	-	-	1	A
49DA	-	+	+	-	-	-	-	-	-	-	-	-	2	A
49DY	-	-	-	-	-	-	-	-	-	-	-	-	0	A
50DA	-	-	+	-	-	-	-	-	-	-	-	-	1	A
51DA	-	-	+	-	-	-	-	-	-	-	-	-	1	A
53DA	-	+	+	-	-	-	-	-	-	-	-	-	2	A
54DA	-	-	+	-	-	-	-	-	-	-	-	-	1	A
56DA	-	-	-	-	-	-	-	-	-	-	-	-	0	A
57DA	-	-	+	-	-	-	-	-	-	-	-	-	1	A
58DA	-	-	+	-	-	-	-	-	-	-	-	-	1	A
63DA	-	+	+	-	-	-	-	-	-	-	-	-	2	A

-=Absence of expected amplicon, +=Presence of expected amplicon, DA=Adult duck, DY=Young 560 duck, Virulence profile A=Presence of 0-2 virulence genes, Profile B=Presence of 3-5 virulence genes. AFEC=Avian fecal *Escherichia coli*

The frequency of the virulence genes in duck isolates is shown in Table-1. The most prevalent virulence-associated genes in the duck AFEC isolates tested were the *fimH* (92.5%), *sit D* (29.6%), and *frz* (3.7%). Relating age and presence of virulence genes, the results showed that there was no link between the age of the poultry species and the presence of virulence genes as isolates from both the young and adult species contained virulence genes.

### Plasmid profiling

Plasmid profiling was done for 45 AFEC isolates using band sizes for the plasmid DNA isolated from the AFEC isolates. The study identified four different profiles A, B, C, and D (Table-4). The most prevalent plasmid profile was profile A (84.4%). In the study, four isolates did not harbor plasmids.

**Table-4:** Plasmid content and profiles in AFEC from turkeys, geese and ducks.

Poultry species	Isolate number	Plasmid content (kb)	Plasmid profile
Turkeys	1TA	55	A
	4TA	55	A
	6TA	55	A
	7TY	55	A
	8TY	55	A
	9TY	55	A
Geese	5GA	55	A
	6GA	55	A
	7GA	-	-
	8GA	55	A
	12GA	55	A
	18GA	55	A
	19GA	55	A
	22GA	-	-
	25	-	-
	27GA	55	A
	28GA	55	A
	29GA	55	A
Ducks	4DA	55	A
	7DA	55	A
	12DA	55	A
	15DA	55	A
	21DA	55	A
	23DA	55	A
	23DY	55	A
	26DY	55; 3.5	B
	31DY	55	A
	32DA	55; 4.5; 1.5	C
	33DA	55	A
	34DA	55	A
	35DA	55	A
	37DA	55	A
	39DA	55	A
	46DA	55	A
	48DA	55	A
	49DA	55	A
	49DY	55	A
	50DA	55	A
	51DA	55	A
	53DA	55	A
	54DA	55	A
	56DA	55	A
	57DA	-	-
	58DA	55	A
	63DA	55; 5	D

AFEC=Avian fecal *Escherechia coli*

### Phylogenetic analysis

**Figure-2:**
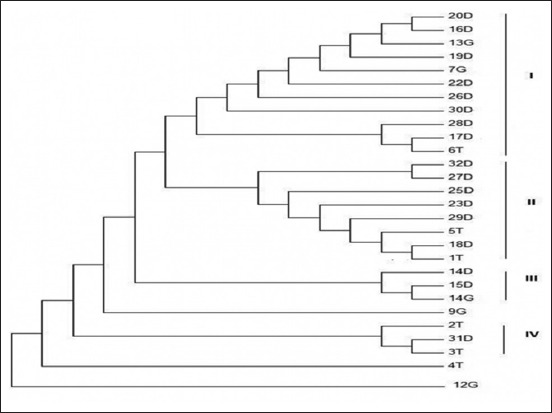
Phylogenetic analysis of avian fecal *Escherichia coli* isolates using maximum likelihood method.

A total of 33 sequences were sent for sequencing, and after editing, 28 were used for phylogenetic analysis. Phylogenetic reconstruction of the 16S rRNA sequences was done using MEGA7 and yielded the phylogenetic tree shown in Figure-2. The evolutionary history was inferred using the maximum likelihood method (supporting bootstrap values from 100 replicates) based on the Tamura-Nei model [19]. The analysis involved all 28 sequences obtained in this study. The derived phylogenetic tree had four clusters. The clusters showed intermingling of AFEC from different avian species. Of the 28 isolates, 11 belonged to cluster I, 8 to cluster II, 3 to cluster III, and 3 to cluster IV, the other 3 isolates were outliers. The AFEC isolates from turkeys, geese, and ducks were randomly distributed and showed intermingling among the four clusters showing that the isolates are closely related to each other regardless of the avian species.

## Discussion

Antimicrobial resistance is a global problem that is affecting both human and veterinary medicine [20]. High resistance of *E. coli* strains should arouse concern as the avian species enter the food chain and thus pose a problem of food safety [21]. The increase in the number of organisms that are resistant to antibiotics is attributed to the over usage of antibiotics as food supplements, prophylaxis and growth promoters over the years [22,23]. Tabatabaei and Nasirian [24] stated that exposing poultry to antimicrobial agents has provided a selection pressure for resistant bacteria. The current study showed that all 54 AFEC isolates exhibited MDR as the isolates were resistant to 2 or more antimicrobial agents (Table-2). Complete resistance was observed for all isolates for the antibiotics bacitracin and cloxacillin (100%) (Figure-1). High resistance was also observed for ampicillin. A study in Malaysia indicated that 72.7% of the AFEC isolates were resistant to Ampicillin [25]. The beta-lactam antibiotics have been used for a very long time in the poultry industry; hence, *E. coli* strains have acquired resistance strategies to this class of antibiotics such as beta-lactamases. Penicillins and beta-lactamase inhibitors are important groups of antimicrobial agents for the treatment of diseases in veterinary medicine and animal food production. The sub-therapeutic dosage used in animal feed accounts for the emergence of resistant strains [22]. In another study, 86.5 % of the AFEC isolates were resistant to one or more antimicrobial drugs [24]. The AFEC isolates from all the three poultry species were susceptible to ciprofloxacin; this is similar to results obtained by Sayah *et al*. [26]. In the study, *E. coli* isolates obtained from both adult and young poultry species (duckling and poults), exhibited MDR thus showing possible cross-contamination between adult and young poultry species.

Although research has increasingly focused on the pathogenesis of APEC infections and the “APEC pathotype,” we have little knowledge about the reservoir of these bacteria, considerably hampering disease control [27]. Virulence genotyping has been suggested to be the best way to differentiate APEC from AFEC, while other typing methods, especially serotyping, might not be particularly useful due to the overlap in serogroups not only between APEC and fecal isolates but also between APEC and other ExPEC isolates [27]. Multiplex PCR technique is capable of identifying the most highly pathogenic *E. coli* isolates in a flock. In the present study, 45 AFEC isolates were analyzed for virulence genes using multiplex PCR. The isolates from turkeys and geese did not contain any virulence genes. The results in Table-3 show that most of the tested isolates from ducks had virulence genes, with 26 (96.3%) of the isolates being classified in profile A (0-2 virulence genes) and 1 isolate (3.7%) exhibiting profile B (3-5 genes) (Table-3). The results showed that the virulence genes are rarely all present in the same isolate thus showing they can occur individually or polygenically [13]. The finding may mean that the isolates from turkeys and geese might have been true commensals which have not yet evolved to possess virulence traits [1].

The few numbers of virulence genes obtained for the isolates is in congruence with other studies that have shown that the AFEC samples have fewer virulence genes compared to APEC [20,28]. Several studies have revealed that significant differences in the distribution of virulence factors among APEC strains and AFEC, suggesting that the APEC strains are well adapted to a pathogenic lifestyle [1,29]. The results (Table-3) show that 92.5% (25/27) of the AFEC from ducks contained virulence genes. The distribution of the virulence genes can be linked to mobile genetic elements which mediate horizontal gene transfer of genes between or among species [30]. In the current study, a fimbrial gene (*fim*H) was found in 92.2 % of the isolates, the *sitD* gene with a prevalence of 29.6 % and *frz* with 3.7%, the rest of the genes from the study showed 0% prevalence (Table-1). The study showed a 29.6% prevalence of the *sitD* gene (Table-1). This finding was a bit higher than that recorded in a study by Mbanga and Nyararai [13] on APEC.

SitABCD has been shown to contribute to the virulence of APEC x7122 in the chicken infection model and also to mediate resistance to oxidative stress. The ability of pathogenic bacteria to sequester iron from body fluids is considered pivotal for virulence, and in APEC, this characteristic has been linked with lethality for 1-day-old chicks [31].

In the current study, the type 1 fimbrial adhesion gene (*fim*H) had the highest prevalence in duck AFEC isolates of 92.2% (Table-1). Interestingly, this finding is similar to that of studies on APEC who recorded high occurrences of the *fimH* gene [13,32]. A study by Van der Westhuizen and Bragg [17] found that on all 10 Zimbabwean APEC isolates they worked on, the *fim*H gene was present in all 10 isolates. The *fim*H gene belongs to fimbrial adhesins virulence factors. Fimbriae are proteinaceous filaments or appendages expressed on the bacterial surface that is believed to mediate adherence to host cells [32]. The high prevalence of the *fimH* gene can be explained by the fact that the gene has been reported to occur in commensal *E. coli* as a fitness trait which helps the *E. coli* isolates to colonize the intestinal region [1,2]. Thus it can be suggested that it is normal to find it in AFEC isolates. On the other hand, APEC isolates can bind to avian tracheal cells and Type I fimbriae confer this ability. A study on APEC showed a 94% prevalence of *fimH* gene [33]. The *fimH* is claimed to be responsible for the first step in the colonization of the lungs and air sacs of birds. This indicates that *fimH* has an important role in the pathogenesis of avian colibacillosis [33].

The *fim*H has been shown to contribute to virulence but is new in the diagnostic context [17]. The presence of *sitD*, *fim*H, and *frz* genes further substantiate that these AFEC isolates in the study could be reservoirs of APEC.

Virulence gene profiles were assigned to all AFEC isolates used in the study (Table-3). The AFEC isolates were profiled as 96.3% fitting profile A and 3.7% fitting profile B. The highest number of virulence gene per isolate was 3 genes. These findings are in congruency with those of Van der Westhuizen and Bragg [17] and Mbanga and Nyararai [13] who found that Zimbabwean APEC isolates have fewer virulence genes. This finding and other findings alluded to earlier, strongly suggests that the duck AFEC isolates are potential reservoirs of APEC isolates.

Since ExPEC that cause clinical disease are thought to emerge from the fecal microbiota of healthy hosts, it is plausible that some commensal intestinal *E. coli* could also harbor large, transmissible plasmids conferring a multidrug-resistant phenotype [20]. Comparison of plasmid profiles is a useful method for the assessment of the possible relatedness of individual clinical isolates of a particular bacteria species for epidemiological studies [34]. Most of the isolates harbored a plasmid assumed to be more than 50 kb as recorded in some literature [35,36].

The present study reported the occurrence of mostly the 55 kb plasmid and other smaller plasmids which are <10 kb. These 55 kb plasmids and the other small plasmids have been shown to be associated with antibiotic resistance [37]. A study by Wu [38] found that the 55 kb plasmid in *E. coli* is linked to antibiotic resistance of antibiotic drugs such as ampicillin, chloramphenicol, streptomycin, and trimethoprim.

Although disagreement exists regarding to what extent multidrug-resistant, poultry-associated strains have emerged and are persisting due to antimicrobial usage in the poultry production environment, it is evident that MDR is now widespread in *E. coli* of poultry origin and is assumed to be associated with conjugative plasmids. A study on the same AFEC isolates used for the current study reported that the isolates exhibited MDR, and thus, the presence of the plasmids can be used to explain the MDR observed. It is of significant public health concern that multidrug-resistant commensal *E. coli* strains may constitute a potential reservoir of resistance plasmids that could be transferred to pathogenic bacteria [37].

Relationships among organisms are often presented as evolutionary trees. The resolution obtained in a given phylogenetic tree is generally thought to be a schematic picture of our understanding of the evolutionary history of these organisms. A group of species appears as a clade when they all descend from a node from which no other species that is not a member of this group also descend [39,40]. The current study detected four clusters for the 28 isolates of which no particular cluster had isolates from one avian species (Figure-2). Cluster 1 was dominated by AFEC from ducks, cluster IV was dominated by AFEC from turkeys. The distribution of the AFEC species in the clusters suggests that there is a close relationship among the AFEC isolates from turkeys, geese, and ducks. The close relationship can be accounted for by the fact that the AFEC isolates in the study were taken from the same farm and hence may possess the same genetic make- up. The study also showed that there were 3 outliers, 2 geese AFEC isolates and 1 turkey AFEC isolates. Direct amplification and sequencing of 16S genes provides a more representative view of a microbial community structure than classical pure culture techniques and has more discriminatory power [39]. The analysis of phylogenetic groups along with detection of virulence genes provides a useful tool for predicting potential health risks associated with *E. coli* [40].

## Recommendations

A more definite conclusion can be obtained using a larger sample size, with the samples being obtained from different geographical locations in the country to eliminate bias as geographical location of strains affects the distribution of virulence traits and other genetic elements. To ascertain that AFEC are reservoirs of APEC, clinical studies and *in vivo* tests should be done using the isolates to determine their pathogenicity. A study on the antibiotic resistance genes can be done to check if the same genes occur in isolates which share multidrug resistance patterns.

## Conclusion

The study revealed that multidrug resistance is high in AFEC isolated from healthy birds. This is a public health concern as the birds are a food source that is potentially contributing to the marked increase in antibiotic resistance in human populations. The study also revealed that of the 3 avian species, the isolates from ducks had virulence genes and thus could be potential reservoirs of APEC. The plasmids that were harbored by the isolates were most likely antibiotic resistance plasmids based on size. The phylogenetic tree showed that the AFEC isolates from turkeys, geese, and ducks are closely related. The clusters showed intermingling of the AFEC isolates suggesting there could be transfer of AFEC isolates between the different poultry species.

## Authors’ Contributions

This paper is extracted from Master of Applied Microbiology and Biotechnology thesis in which ND was the graduate student who carried out experimental work and wrote the thesis. JM was the supervisor of the research and participated in project design, organization of data analysis, and design of the manuscript. All authors read and approved the manuscript.
